# Automatic electronic reporting improved the completeness of AMI and stroke incident surveillance in Tianjin, China: a modeling study

**DOI:** 10.1186/s12963-023-00300-2

**Published:** 2023-02-06

**Authors:** Hong Xiao, Fang Liu, Joseph M. Unger

**Affiliations:** 1 Public Health Sciences Division, Fred Hutchinson Cancer Center, Seattle, WA USA; 2 Chinese Center for Disease Control and Prevention, Beijing, China

**Keywords:** Stroke, Acute myocardial infarction, DisMod, Incidence surveillance

## Abstract

**Background:**

AMI and stroke are the leading causes of premature mortality and hospitalizations in China. Incidence data at the population level for the two diseases is limited and the reliability and completeness of the existing incidence registry have not been investigated. We aim to assess if the completeness of case ascertainment of AMI and stroke incidence has improved since the implementation of electronic reporting and to estimate the incidence of AMI and stroke in Tianjin, China.

**Methods:**

We applied the DisMod II program to model the incidence of AMI and stroke from other epidemiological indicators. Inputs include mortality rates from Tianjin’s mortality surveillance system, and the point prevalence, remission rates and relative risks taken from IHME’s Global Burden of Disease studies. The completeness of AMI and stroke incidence reporting was assessed by comparing the sex and age-specific incidence rates derived from the incidence surveillance system with the modeled incidence rates.

**Results:**

The age and sex standardized modeled incidence per 100,000 person-year decreased (*p* < 0.0001) from 138 in 2007 to 119 in 2015 for AMI and increased (*p* < 0.0001) from 520 in 2007 to 534 in 2015 for stroke. The overall completeness of incidence report was 36% (95% CI 35–38%) for AMI and 54% (95% CI 53–55%) for stroke. The completeness was higher in men than in women for both AMI (42% vs 30%, *p* < 0.0001) and stroke (55% vs 53%, *p* < 0.0001) and was higher in residents aged 30–59 than those aged 60 or older for AMI (57% vs 38%, *p* < 0.0001). The completeness of reporting increased by 7.2 (95% CI 4.6–9.7) and 15.7 (95% CI 14.4–16.9) percentage points for AMI and stroke, respectively, from 2007 to 2015 among those aged 30 or above. The increases were observed in both men and women (*p* < 0.0001) and were more profound (*p* < 0.0001) among those aged between 30 and 59 and occurred primarily during the 2010 and 2015 period.

**Conclusions:**

Completeness of AMI and stroke incidence surveillance was low in Tianjin but has improved in recent years primarily owing to the incorporation of an automatic reporting component into the information systems of health facilities.

**Supplementary Information:**

The online version contains supplementary material available at 10.1186/s12963-023-00300-2.

## Introduction

Reliable estimations of representative population-level disease incidence are crucial for the prioritization of health service planning and policy making, and provide important indicators for assessing the effectiveness of preventive measures and management of risk factors. Such estimates also provide essential inputs for the calculation of the Disability-Adjusted Life Year, a vital metric for the evaluation of burden of diseases [1]. Estimates of epidemiological data come from different sources: mortality data are typically derived from civil registration and vital statistics, while incidence data are from disease registry or population-level cohort studies [2]. For many diseases, incidence is often more difficult to measure than mortality due to substantial incompleteness and significant internal inconsistency [3]. Lack of consistency in epidemiology estimates, an indicator for potential measurement error, exists even within a well-defined population [2].

AMI and stroke are major causes of hospitalization and the leading causes of premature mortality [4, 5] in China. Given the increasing burden of AMI and stroke, it is essential to monitor their incidence. Empirical data on the incidence of the two diseases at either national or sub-national levels are limited. Existing data sources do not yield sufficient and long-term continuous AMI incidence information [6–9]. For instance, the China Acute Myocardial Infarction Registry covered over 100 hospitals from all provinces and municipalities throughout Mainland China. The registry, which obtains clinical characteristics, diagnosis, treatment and outcomes of Chinese AMI patients, may not provide AMI incidence estimates that fully represent what occurs at the population level [4].

Tianjin, the third largest city (population 15.6 million) in China, has been routinely collecting incidence data of major non-communicable diseases (NCDs) including AMI and stroke through the Incidence Surveillance System established in 1984. In 2007, Tianjin incorporated the NCDs Incidence Surveillance component into the Hospital Information System (HIS) of pilot hospitals, reporting new cases directly via HIS rather than exclusively relying on conventional manually reporting cards. The derived incidence estimates have been utilized in burden of disease studies, despite the fact that the quality of the data—including evaluations of data completeness and consistency with external datasets or other relevant epidemiological indicators—has not be investigated [10–12].

Originally developed for the Global Burden of Disease studies, DisMod II has been extensively used to supplement observational data and assess internal consistency [2]. DisMod II is a multistate life table that describes the transitions between the disease states “health”, “diseased” and “dead” by using the transition rates from incidence to remission to case fatality [13]. By solving a set of linear differential equations, DisMod II can estimate age-specific incidence as well as prevalence of a disease given sufficient data on other disease variables. Compared with DisMod, an Incidence–Prevalence–Mortality model that requires three transition hazards incidence (from health to disease), remission (from disease to health) and case fatality (from disease to cause-specific death) as inputs, DisMod II allows for a wider range of input variables including incidence as a population rate, prevalence, duration, and mortality. Further, the application of DisMod II in estimating the incidence of a disease does not require multiple independent incidence data sources as prerequisites, a limitation inherent in the capture-recapture approach.

In this study, we examined the completeness and consistency of the reported AMI and stroke incidence rate by comparing the observed rates with model-based predicted incidence rates, and assessed if the completeness of reported incidence in Tianjin has improved since the introduction of the direct automatic reporting through HIS.

## Methods

### Data

#### Incidence rate

The age- and sex specific incidence rate data have been published elsewhere and are publicly available [10, 14, 15]. Incidence cases in published datasets were originally extracted from the Incidence Surveillance System in Tianjin, which was initially launched for a community-based program on the prevention and control of major NCDs including AMI and stroke [12]. The study subjects are residential registered cases who have experienced AMI or stroke, diagnosed by a hospital or clinic in 2007, 2010 or 2015. Cases were included in the dataset if they met all the following inclusion criteria:

1. Stroke detected and diagnosed by a medical practitioner within 28 days of onset;
2. AMI/stroke onset within the study period. Recurrence within 28 days was not included in incidence calculations, and recurrence after 28 days was considered as a new case;
3. Registered permanent (Hukou) resident of Tianjin at the time of AMI/stroke onset. Hukou based registration a household registration record officially identifies a person as a permanent resident of an area and includes identifying information such as name, parents, spouse and date of birth.

The recording process and quality control of the data have been described elsewhere [16]. New case reporting cards were required to be completed or entered into the Non-Communicable Disease Incidence Surveillance System by clinicians in hospitals or community clinics in Tianjin. The records include gender, date of birth, the diagnosis and diagnostic basis, date of events, job type, insurance status, smoking status, and district of residence. In 2007, the Tianjin CDC (Center for Disease Control and Prevention) incorporated the NCDs incidence surveillance component into the HIS of pilot hospitals [17]. By 2015, over 70% of the new cases from the chronic non-communicable incidence surveillance system were reported directly via HIS [18]. New cases in the surveillance system, either reported by cards or via HIS, were verified through various methods including regular training for doctors and checking daily reports for recurrence, logical errors and codes on a case-by-case basis at three levels (hospital, district and municipal CDC).

Incidence classification was coded in accordance with the International Classification of Disease, 10th Edition (ICD-10). The diseases used as incidence outcome measures in this study are AMI (ICD-10, I21) and stroke (ICD-10, I60-I64). Definitions included patients with symptoms, and imaging, laboratory and clinical examinations. Hemorrhagic stroke was defined as a stroke event with the diagnosis of subarachnoid hemorrhage or intracerebral hemorrhage, and ischemic stroke was defined as a stroke event with the diagnosis of thrombosis or embolism (hemorrhagic stroke ICD-10: I60, I61, I62; ischemic stroke: I63).

#### Cause-specific mortality rate

The age- and sex specific mortality rate of AMI and stroke for the year 2007, 2010 and 2015 were extracted from published reports and datasets [15, 19]. The original data were obtained from the all-cause mortality surveillance system, which monitors the entire residential population of the city. Deaths were ascertained through the procedures that have been proposed to be used in the Disease Surveillance Points in China. The recording process and quality control of the data have been described elsewhere [16, 20]. In brief, practicing clinicians from hospitals or community clinical centers completed death certificates and submitted them to the mortality surveillance system. Trained community clinicians (community health workers or village doctors) investigated the underlying causes of non-hospital deaths by interviewing relatives of the deceased and by reviewing available medical records on a door-to-door basis. The district and municipal CDCs oversee and check the quality of death certificates at the primary and secondary levels, respectively. The municipal CDC also provides technical training and support to staff involved in the surveillance process [20]. Cause of death classification for the study period was based on ICD-10.

#### Prevalence, remission rate and relative risk

Data on the prevalence, remission rate and relative risk of AMI and stroke in Tianjin were taken from the Global Burden of Disease (GBD) and Local Burden of Disease (LBD) studies conducted by the Institute for Health Metric and Evaluation (IHME). These epidemiological estimates were modeled using the DisMod-MR 2.0, a Bayesian mixed-effects meta-regression modeling technique developed for IHME’s GBD/LBD studies [21]. The estimates are initially made at the global level then sequentially revised down to the national and subnational levels using progressively more detailed data.

The prevalence, expressed as a proportion, is the number of cases in a population at a moment in time. The remission rate is the number of cases that resolve or are cured per person-year among patients. The incidence rate is the number of new cases per person-year. Stroke events are categorized into four groups in IHME’s dataset: acute/chronic ischemic stroke, and acute/chronic hemorrhage stroke. We used the sum of the prevalence of each stroke subtype as the prevalence of all stroke, and the weighted mean of the remission rates/relative risks as the remission rate/relative risk of all stroke. The percent weight given to each subtype was calculated as its fraction of all stroke prevalence. As the estimates from GBD studies were not produced on a yearly basis, we utilized the 2016 estimates and the mean of the estimates for the year 2005 and 2010 as the approximation for inputs of DisMod II for the year 2015 and 2007, respectively.

#### Population structure

Single-year permanent population estimates by sex and five-year age groups were taken from the Residence Registry Section of the Municipal Bureau of Public Security. We used WHO World Standard Population based on world average population between 2000 and 2025 for the purpose of calculating age standardized rates. Ethical approval for this study is not required because all data sources are publicly available aggregated data.

### Model setting and analysis

**Fig. 1:**
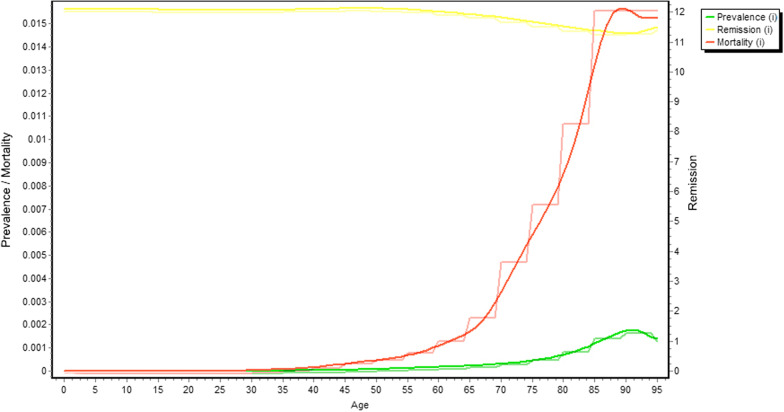
The data inputs for the DisMod II: actual and smoothed AMI prevalence, remission and mortality rate for male in 2007. Relative risk is not presented in the graph given the scale of y-axis

The raw input data and population data were recorded by sex and 5-year age groups. To produce smooth rates for the modeled outputs, DisMod II first interpolates the input data into single age estimates using a cubic spline model for all input data (mortality rate, prevalence, remission rate and relative risk). Figure 1 shows the model input data (both original and smoothed) by age for AMI in men in 2007.

Analyses were conducted with and without accounting for trends in incidence rates. When trends were accounted for, an annual change in the AMI incidence rate of − 2% for both males and females, and an annual change in the stroke incidence rate of 3% and 2% for males and females, respectively, since the year 2000 were incorporated into the models. These trends were based on studies of AMI and stroke trends in Tianjin [10, 12, 22, 23]. We conducted DisMod II uncertainty analysis to assess the 95% confidence interval of the modeled incidence estimates. In the uncertainty analysis, DisMod II conducts a bootstrapping exercise where the input variables are assumed to follow a specific distribution. We allowed the estimates of AMI and stroke prevalence to vary with a normal distribution and set the number of bootstrap iterations to 100. Analyses of uncertainty for models that included trends did not converge under our modeling framework; uncertainty estimates in these cases were not provided.

We calculated the proportion of incidence cases ascertained by the incidence surveillance system (completeness of reporting) as the observed incidence rate divided by the modeled incidence rate accounting for trends (the incidence rate ratio, IRR). We tested if the IRR increased from 2007 to 2010 and 2015 using one-sided Z-test. All the statistical tests were performed with R Version 3.5.3 using a 0.05 significance level. The RECORD (REporting of studies Conducted using Observational Routinely-collected health Data) guidelines were followed. DisMod II analytical software tool is freely available for use and can be downloaded from http://www.epigear.com/index_files/dismod_ii.html. The datasets analyzed in the study are available from the corresponding author upon request.

## Results

23,371 AMI incident cases and 152,394 stroke incident cases were ascertained by Tianjin’s incidence surveillance system for the three years, which constituted an age-standardized incidence rate of 51 (male 65, female 36) and 316 (male 389, female 242) per 100,000 population per year for AMI and stroke, respectively. Overall, 92.4% of the AMI incident cases were diagnosed based on electrocardiography (ECG) and/or imaging examination including ultrasound test and coronary angiography (CAG), and 93.7% of the stroke incident cases were diagnosed based on CT or MRI scanning. The overall observed AMI incidence to mortality rate ratio was 0.90 (95% CI 0.88–0.91) from 2007 to 2015, and observed stroke incidence to mortality rate ratio increased significantly from 2.90 (95% CI 2.84–2.96) in 2007 to 7.71 (95% CI 7.54–7.90) in 2015 (Additional file 1: Figure S1).

Table 1 shows that AMI and stroke incidence rates, both reported and modeled, are higher (*p* < 0.0001) in men than in women, and are higher (*p* < 0.0001) in older age groups than in younger age groups. The observed AMI incidence rate showed a monotonic downward trend from 2007 to 2015 for both men and women, and for the majority of age groups. The observed stroke incidence rate showed a monotonic upward trend for both men and women, and for all the age groups, with the upward trend becoming more pronounced after 2010. An overall decrease in modeled AMI incidence and increase in modeled stroke incidence rates, including estimates that both did and did not account for temporal trends, were found among men and women for the majority of age groups. The age and sex standardized modeled incidence per 100,000 person-years decreased (*p* < 0.0001) from 138 (95% CI 125–145) in 2007 to 119 (95% CI 113–125) in 2015 for AMI and increased (*p* < 0.0001) from 520 (95% CI 450–582) in 2007 to 534 (95% CI 534–618) in 2015 for stroke.

**Table 1:** Number and rate of AMI/stroke incidence in Tianjin, 2007, 2010 and 2015

	Number									Incidence rate** per 100, 000 (95% confidence interval)								
	2007			2010			2015			2007			2010			2015		
	Observed	DisMod II	DisMod II*	Observed	DisMod II	DisMod II*	Observed	DisMod II	DisMod II*	Observed	DisMod II	DisMod II*	Observed	DisMod II	DisMod II*	Observed	DisMod II	DisMod II*
AMI																		
Men																		
0–29	21	203	200	16	229	226	16	218	215	1 (0, 1)	12 (11, 13)	12	1 (0,1)	13 (11, 14)	13	1 (0, 1)	13 (11, 14)	13
30–64	1634	2560	2527	1738	2846	2806	1950	2787	2751	61 (58, 65)	96 (87, 105)	95	61 (587 64)	98 (87, 106)	97	69 (65, 72)	96 (87, 102)	95
65+	2870	6548	6487	2956	7954	7800	3391	7722	7649	380 (364, 396)	880 (808, 917)	872	344 (322, 347)	917 (840, 976)	901	311 (292, 316)	670 (616, 697)	705
Total	4525	9311	9215	4710	11,029	10,832	5357	10,727	10,615	69 (66, 71)	147 (134, 155)	146	64 (62, 66)	163 (139, 163)	150	63 (61, 65)	122 (112, 129)	126
Women																		
0–29	3	160	158	4	212	210	3	204	202	1 (0,1)	10 (9, 12)	10	1 (0, 1)	13 (12, 14)	13	1 (0, 1)	13 (12, 14)	13
30–64	380	1298	1283	484	1243	1225	452	1001	987	14 (12, 16)	50,944, 53)	49	16 (15, 18)	43 (38, 47)	43	15 (13, 17)	34 (30, 37)	34
65+	2365	7220	7171	2405	9366	9289	2683	9104	9010	283 (269, 297)	869 (798, 922)	863	252 (239, 265)	952 (875, 1002)	944	236 (224, 249)	687 (624, 727)	751
Total	2748	8678	8612	2893	10,821	10,724	3138	10,309	10,199	39 (37, 41)	128 (116, 136)	127	36 (35, 38)	137 (125, 145)	135	34 (32, 35)	102 (92, 108)	109
Stroke																		
Men																		
0–29	50	866	1086	65	787	908	129	600	682	2 (1, 3)	47 (40, 52)	57	3 (2, 3)	40 (33, 48)	45	5 (4, 6)	34 (29, 38)	38
30–64	7978	13,660	16,114	9488	15,276	16,729	13,210	15,388	16,928	294 (286, 302)	514 (453, 576)	608	320 (311, 328)	526 (470, 585)	577	453 (444, 463)	528 (460, 595)	582
65+	14,863	25,470	29,665	17,399	29,442	31,992	25,944	38,899	42,717	1965 (1929, 2001)	3384 (3038, 674)	3943	2040 (2004, 2076)	3444 (3066, 3796)	3743	2396 (2358, 2435)	3534 (3075, 3939)	3950
Total	22,891	39,995	46,865	29,364	45,505	49,629	39,283	54,888	60,327	345 (340, 351)	620 (552, 680)	727	364 (359, 370)	628 (558, 696)	685	458 (452, 464)	636 (554, 709)	708
Women																		
0–29	31	358	381	30	432	467	63	421	450	1 (1, 2)	23 (16, 30)	24	1 (1, 2)	26 (17, 32)	28	3 (2, 3)	27 (23, 30)	29
30–64	3948	10,106	10,713	4505	11,130	11,884	6015	9847	10,548	145 (140, 151)	381 (308, 447)	404	148 (142, 153)	386 (311, 464)	412	202 (195, 208)	338 (250, 420)	367
65+	10,990	18,782	20,002	13,803	21,719	23,323	19,575	27,975	30,392	1343 (1313, 1373)	2227 (1881, 2526)	2442	1492 (1461, 1524)	2293 (1937, 2603)	2493	1686 (1653, 1720)	2324 (1802, 2829)	2581
Total	14,969	29,246	31,096	20,234	33,281	35,674	25,653	38,244	41,389	215 (211, 220)	420 (348, 483)	455	234 (230, 238)	428 (355, 493)	466	278 (273, 283)	418 (320, 510)	458

*With trend

**Standardized incidence rate

**Fig. 2:**
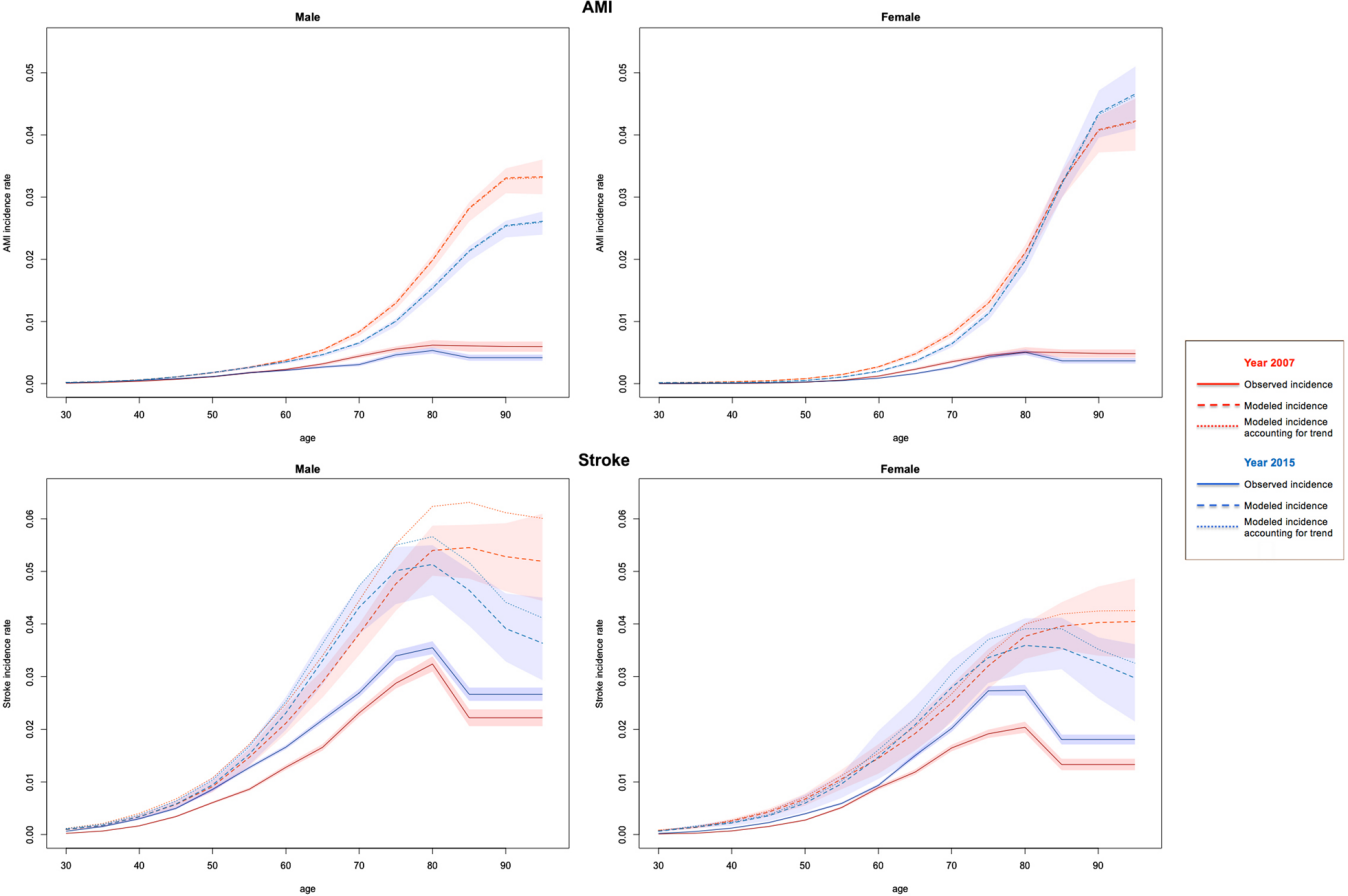
Age specific incidence rates of AMI and stroke in Tianjin, 2007 and 2015. Note: The line for the modeled AMI incidence and that for the modeled AMI incidence accounting for trends virtually overlap

Figure 2 presents the age-specific observed and modeled incidence rates for AMI and stroke in 2007 and 2015. The figures show that the reported incidence rates are significantly lower than the modeled estimates for both men and women. The trended estimates from DisMod II for stroke incidence is higher than the non-trended estimates. The overall ratio of observed to modeled incidence was approximately 38.8% and 57.5% for AMI and stroke, respectively. Table 2 presents the sex and age-group specific ratio of observed to modeled incidence rate, both trended and non-trended estimates, for AMI and stroke. For AMI, there is no significant difference between trended and non-trended ratios, for either men or women, and either younger or older groups. While for stroke, the trended ratio is lower (*p* < 0.05) than non-trended one for both men and women, and both younger and older groups. The trended ratio for AMI increased significantly from 2007 to 2015 in the 30–59 age group for both men (0.64 vs 0.73, *p* = 0.026) and women (0.29 vs 0.44, *p* < 0.0001). The trended ratio for stroke increased in both the younger (30–59) age group (0.48 verse 0.78 for men, and 0.36 verse 0.55 for women, all *p* < 0.0001), and the older (≥ 60) age group (0.50 verse 0.61 for men, and 0.55 verse 0.65 for women, all *p* < 0.0001).

**Table 2:** Ratio of reported to modeled* incidence rate of AMI and stroke in Tianjin, 2007, 2010 and 2015

	2007		2010		2015	
	Male	Female	Male	Female	Male	Female
AMI						
0–29	0.07 (0.05, 0.11)	0.01 (0.00, 0.04)	0.05 (0.03, 0.09)	0.01 (0.01, 0.03)	0.05 (0.03, 0.08)	0.01 (0.00, 0.03)
30–60	0.64 (0.59, 0.69)	0.29 (0.25, 0.33)	0.62 (0.57, 0.67)	0.38 (0.33, 0.43)	0.72 (0.67, 0.77)	0.43 (0.37, 0.50)
60+	0.43 (0.42, 0.45)	0.33 (0.31, 0.35)	0.38 (0.36, 0.40)	0.26 (0.25, 0.28)	0.44 (0.41, 0.46)	0.31 (0.29, 0.33)
Total	0.47 (0.45, 0.49)	0.31 (0.29, 0.33)	0.42 (0.40, 0.44)	0.27 (0.25, 0.28)	0.50 (0.48, 0.52)	0.31 (0.29, 0.32)
AMI*						
0–29	0.07 (0.05, 0.12)	0.01 (01, 0.04)	0.05 (0.03, 0.09)	0.01 (0.01, 0.03)	0.05 (0.03, 0.08)	0.01 (0.00, 0.03)
30–60	0.64 (0.60, 0.69)	0.29 (0.25, 0.33)	0.63 (0.58, 0.68)	0.38 (0.34, 0.44)	0.73 (0.68, 0.78)	0.44 (0.38, 0.51)
60+	0.44 (0.41, 0.46)	0.33 (0.31, 0.35)	0.38 (0.36, 0.40)	0.27 (0.25, 0.28)	0.44 (0.42, 0.47)	0.31 (0.30, 0.33)
Total	0.47 (0.45, 0.49)	0.31 (0.29, 0.33)	0.43 (0.41, 0.45)	0.27 (0.25, 0.28)	0.5 (0.48, 0.52)	0.31 (0.29, 0.33)
Stroke						
0–29	0.05 (0.04, 0.06)	0.06 (0.04, 0.09)	0.06 (0.05, 0.08)	0.04 (0.03, 0.06)	0.15 (0.13, 0.18)	0.10 (0.08, 0.13)
30–60	0.57 (0.55, 0.59)	0.38 (0.36, 0.40)	0.61 (0.59, 0.63)	0.38 (0.37, 0.40)	0.86 (0.83, 0.88)	0.60 (057, 0.62)
60+	0.58 (0.57, 0.59)	0.59 (0.57, 0.60)	0.59 (0.58, 0.61)	0.64 (0.63, 0.66)	0.67 (0.65, 0.68)	0.71 (0.69, 0.73)
Total	0.56 (0.55, 0.57)	0.5 (0.49, 0.52)	0.58 (0.57, 0.59)	0.54 (0.53, 0.55)	0.71 (0.70, 0.72)	0.66 (0.64, 0.67)
Stroke*						
0–29	0.04 (0.03, 0.05)	0.06 (0.04, 0.08)	0.06 (0.04, 0.07)	0.04 (0.03, 0.06)	0.14 (0.11, 0.16)	0.09 (0.07, 0.13)
30–60	0.48 (0.47, 0.50)	0.36 (0.34, 0.38)	0.55 (0.54, 0.57)	0.36 (0.34, 0.37)	0.78 (0.76, 0.80)	0.55 (0.53, 0.57)
60+	0.50 (0.49, 0.51)	0.55 (0.53, 0.57)	0.55 (0.53, 0.56)	0.6 (0.58, 0.61)	0.61 (0.59, 0.62)	0.65 (0.64, 0.67)
Total	0.48 (0.47, 0.48)	0.47 (0.46, 0.48)	0.53 (0.52, 0.54)	0.50 (0.49, 0.51)	0.65 (0.64, 0.66)	0.61 (0.59, 0.62)

^*^With trend

**Fig. 3:**
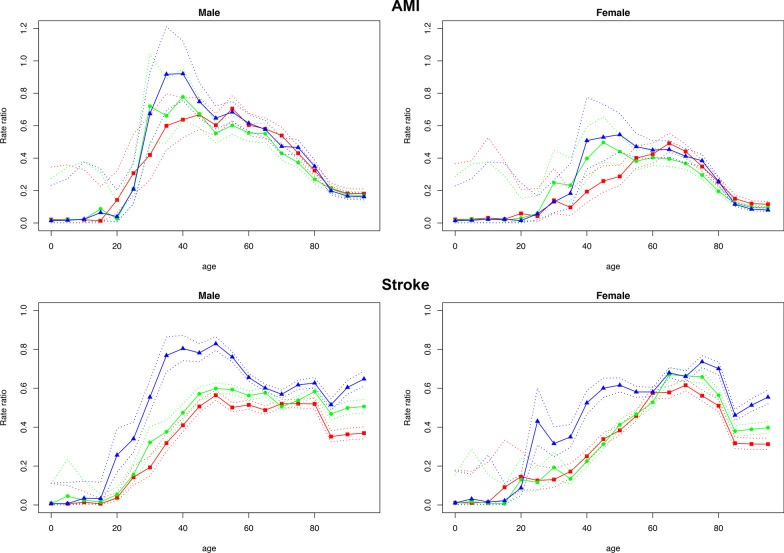
Age-specific ratio of reported to modeled incidence rate (trended) of AMI and stroke by sex and year. Red, green and blue solid lines represent estimates for 2007, 2010 and 2015, respectively. Dashed lines are upper and lower boundaries of the 95% confidence intervals

For those aged 30 years or above, the ratio was higher in men than in women, for both AMI and stroke incidence, and for both younger subgroup (age 30–59) and older subgroup (age ≥ 60), except for stroke incidence among the older age group (age ≥ 60). For the 60-plus age group, the ratio for stroke was consistently higher (*p* < 0.0001) than that for AMI for both men and women. For the 30–59 age group, the ratio for stroke was lower (*p* < 0.0001) in 2007 and 2010, but higher (*p* = 0.058) in 2015 than that for AMI. Figure 3 illustrates the age-specific ratio of reported to modeled incidence rate for AMI and stroke by sex and year. It shows that the increases in the ratio for both AMI and stroke occurred primarily during the 2010 and 2015 period.

## Discussion

This is the first published study that aims to estimate the completeness of routine incidence surveillance for cardiac and cerebrovascular diseases in Tianjin. Our findings indicate that the overall case ascertainment was approximately 39% and 58% for AMI and stroke, respectively. Eliminating trends in the incidence rate of stroke in the DisMod II settings resulted in observed and estimated stroke incidence being less divergent at both younger and older ages than without trends. From 2007 to 2015, an overall increase in the estimated completeness for both AMI and stroke was observed, predominantly attributed to the improvement in the case reporting among the population aged 60 years or younger after the year 2010.

The relatively low overall completeness of routine incidence reporting under passive surveillance in Tianjin could be attributed to several major reasons. First, neither cause-specific mortality data nor healthcare claim data has been linked to the incidence surveillance of the two diseases. Passive incidence registry is often unlikely to capture incident cases that lead to sudden deaths before patients reach health facilities. The average time from AMI symptoms onset to hospital arrival is 4 h in China [24]. In Tianjin, over 70% of AMI and stroke deaths occur at home [25], indicating that successfully identifying and deriving death certificates cases is important to supplement incidence surveillance data. In addition, the incidence registry in Tianjin fails to record incidence cases in instances where healthcare is either not sought, or is accessed in health facilities out of Tianjin. Healthcare claim data, routinely collected by the Municipal Bureau of Human Resources and Social Security, are important to AMI and stroke incidence surveillance as a means of finding cases not captured by local health facilities. Second, patients with minor events may be less likely to seek medical care urgently and are more often coded as a non-specific “other” diagnosis in the administrative database of health facilities. The proportion of patients with minor stroke is higher than reported in even high-quality stroke incidence studies and the underestimation is likely to be much greater in incidence surveillance from low-to-middle income countries, where healthcare utilization is often low [26]. However, it’s impossible to estimating the number of minor cases in Tianjin is problematic since incidence rates stratified by disease severity were not presented in our dataset. Lastly, unlike death certificates that are required by law to be completed and submitted by practicing clinicians from health facilities, AMI and stroke incidence reporting is not mandatory in Tianjin [12]. Although local health workers have been trained to submit accurate and timely case reports, the reporting frequency of health workers and/or facilities has not been continuously monitored. The passive surveillance in Tianjin relies heavily on an extensive network and the cooperation of health workers at all levels of facilities. It is difficult to assure the completeness and timeliness of data submission without individualized feedback or incentives for health workers and health facilities. In their absence, the distributions of monthly incident cases by health facility appear to demonstrate spurious fluctuations and zero reporting during the study period [25].

Our results suggest that the estimated completeness of reporting of AMI and stroke incidence was lower in female and older population, which could, in part, be explained by behavioral, diagnostic and pathological factors. The age of patients in China is negatively associated with the decision to seek health care [27, 28], likely owing to limited access to healthcare services and health insurance availability. Women in China are less likely to seek health care due to higher sensitivity to cost, social power relations and inherent inequality [27, 29]. It has been reported that un-witnessed sudden cardiac death is more likely to occur among women and older people [30] due to unusual pathophysiological mechanisms [31, 32], lower awareness of the warning signs and symptoms [33] and a higher chance of being unemployed and/or living alone. This hypothesis is supported by evidence that a higher proportion of AMI and stroke deaths for women and older people in Tianjin occur at home [25]. In addition, women have less typical symptoms than men and women with minor stroke or transient ischemic attack are less likely to be diagnosed with a stroke despite having similar symptoms at presentation [34].

The improvement in the estimated completeness could be attributed to several factors. First, Tianjin started to incorporate NCDs Incidence Surveillance program into the HIS of some pilot hospitals in 2007, before which new incident cases were exclusively reported through manually completed paper cards. By 2015, over 70% of new cases from the chronic non-communicable incidence surveillance system were reported directly via HIS. The automatic reporting component embedded in HIS significantly reduced the workload of health workers, improving the timeliness and continuity of routine case reporting. Second, out-of-pocket costs are associated with prolonged time in care-seeking and hospital arrival after the onset of cardiovascular disease in China [24]. China’s health care system reform beginning in 2009 has rapidly expanded health insurance coverage, significantly reducing the share of out-of-pocket expenditure and increasing the accessibility and timely utilization of healthcare services [35]. Increased access to and utilization of healthcare services allows the primary care computer systems to systematically identify patients with cardiovascular and cerebrovascular disease [36].

Extensively implemented secondary and tertiary prevention programs and campaigns in recent years may have further increased the likelihood of AMI and stroke patients being captured by the surveillance system. In 2011, Tianjin initiated the Stroke Screening and Prevention Project that screens for eight major risk factors of stroke and recommends labs and imaging tests to be performed on the basis of personal risk estimation [37, 38]. This proactive community-based free screening program has enhanced the public’s awareness of the warning signs and symptoms of stroke, resulting in more timely and appropriate health-seeking decisions. Consequently, the screening project has increased the chance of being diagnosed and coded as having stroke for patients at high risk and patients with minor stroke events [39]. Since 2014, Tianjin has gradually established chest pain centers and a chest pain rescue network that aims to optimize the diagnosis and treatment processes for patients with acute chest pain, especially AMI [40]. Increased access to urgent treatment and reduced hospital arrival time would lower the proportion of uncaptured incident patients who die at home or on the way to healthcare facilities. However, linking cause-specific mortality to the incidence surveillance of the two diseases is warranted to ascertain patients that were missed in the information system of health care facilities.

This study has limitations. First, we assumed that the estimate from DisMod II reflects the true incidence rate and is the “gold standard” for measuring the completeness of reported incident events. Results from some previous studies have shown that the DisMod II estimates for both men and women were very similar to estimates derived from the routine health information system or population health survey [3, 41]. Scarborough et al. compared DisMod II estimates of age-specific incidence rates for AMI with those observed in the external dataset from England [42]. The DisMod II model estimates were unable to replicate age-specific incidence rates of AMI derived from a population-based study, although they were of similar magnitude. Our estimate of the completeness of case reporting is likely to be biased if DisMod II estimate is an imperfect “gold standard” and the inputs, including mortality rate, for DisMod II are not accurate. Second, the lack of any other rigorous research on the prevalence of AMI/stroke, especially the relative risk of mortality and remission of patients with AMI/stroke in Tianjin, required that we utilize the epidemiological estimates provided by IHME’s mixed-effects meta regression modeling using the DisMod-MR 2.0. The accuracy of these local estimates has not been previously investigated. However, importantly, these estimates are internally consistent with the observed mortality rate derived from local all-cause mortality surveillance. Third, the ideal input data for the DisMod II would be estimates of the increased all-cause mortality for people who have had AMI or stroke. Given the lack of direct measures, we used mortality data where AMI or stroke was indicated as the underlying primary cause of death. This metric does not account for increased mortality risk from other conditions (e.g. increased risk of respiratory disease and peripheral vascular disease) [42]. Lastly, under the constraint of data availability, we were not able to extend the analysis to more recent settings, which may presumably dilute the full impacts of the direct automatic electronic reporting system.

Our model is open to further research and validity investigation. Reliable incidence data from extensive prospective follow-up studies and other independent incidence data sources with information provided at the individual level that could be linked to elements of CDC’s incidence surveillance data to supplement the assessment of completeness of routine reporting. Moreover, alternative prevalence and remission rate data will help to further confirm the validity of our findings.

## Conclusions

Our findings indicate that the reporting of AMI and stroke incidence in Tianjin has been incomplete. However, the completeness of the surveillance has been improving since 2010 primarily owing to the incorporation of an automatic reporting component into the information systems of health facilities, the increase in the utilization of healthcare service and campaigns promoting access to prevention services and timely emergency treatment.

## Supplementary Information

**Additional file 1: Figure S1.** Trend of AMI and stroke incidence to mortality rate ratio in Tianjin, Age ≥ 35. Red line, mortality rate; Blue line, incidence rate; Black solid line, incidence to mortality rate ratio (smoothed).

## Data Availability

The data that supports the findings of this research are available upon reasonable request from the corresponding author.

## Abbreviations

AMI: Acute myocardial infarction
NCDs: Non-communicable diseases
ICD: International Classification of Disease
HIS: Hospital Information System

## References

[1] Saha S (2008). Modelling disease frequency measures in schizophrenia epidemiology. Schizophr Res.

[2] Barendregt JJ, Ott A (2005). Consistency of epidemiologic estimates. Eur J Epidemiol.

[3] Kruijshaar ME, Barendregt JJ, Hoeymans N (2002). The use of models in the estimation of disease epidemiology. Bull World Health Organ.

[4] Xu H (2016). The China Acute Myocardial Infarction (CAMI) Registry: a national long-term registry-research-education integrated platform for exploring acute myocardial infarction in China. Am Heart J.

[5] Zhou M (2019). Mortality, morbidity, and risk factors in China and its provinces, 1990–2017: a systematic analysis for the Global Burden of Disease Study 2017. Lancet.

[6] People's Republic of China-United States Cardiovascular and Cardiopulmonary Epidemiology Research Group (1992). An epidemiological study of cardiovascular and cardiopulmonary disease risk factors in four populations in the People's Republic of China: Baseline report from the P.R.C.-U.S.A. Collaborative Study. Circulation.

[7] Wu ZS (1988). The Sino-MONICA-Beijing Study: report on results between 1984 and 1986. Acta Med Scand Suppl.

[8] Wu Y (2006). Estimation of 10-year risk of fatal and nonfatal ischemic cardiovascular diseases in Chinese adults. Circulation.

[9] Liu J (2004). Predictive value for the Chinese population of the Framingham CHD risk assessment tool compared with the Chinese Multi-Provincial Cohort Study. JAMA.

[10] Wang DZ (2017). Fifteen-year trend in incidence of acute myocardial infarction in Tianjin of China. Chin J Cardiovasc Dis.

[11] Li W (2016). Incidence trends of cervical cancer in Tianjin, 2007–2013. Zhonghua Liu Xing Bing Xue Za Zhi.

[12] Jiang G (2016). Epidemiological transition and distribution of stroke incidence in Tianjin, China, 1988–2010. Public Health.

[13] Barendregt JJ (2003). A generic model for the assessment of disease epidemiology: the computational basis of DisMod II. Popul Health Metr.

[14] Liu Y (2017). Incidence rate estimation of acute myocardial infarction (AMI) for people over 35 years old in Tianjin from 2007 to 2015.

[15] Wei C (2018). Epidemiological characteristics of stroke incidence and mortality in Tianjin 2007–2015. Chin J Prev Med.

[16] Xiao H (2019). Impact of smoke-free legislation on acute myocardial infarction and stroke mortality: Tianjin, China, 2007–2015. Tob Control.

[17] Ji Y (2012). The construction and management of electronic reports for non-communicable diseases. Chin J Prev Control Chronic Dis.

[18] Wang D. Leveraging the surveillance system for NCDs to develop health policy. Tianjin, China; 2016.

[19] Wang DZ (2017). Analysis on the trends in mortality following acute myocardial infarction from 1999 to 2015 in Tianjin of China. Chin J Cardiovasc Dis.

[20] Wang X (2009). Surveillance of trend and distribution of stroke mortality by subtype, age, gender, and geographic areas in Tianjin, China, 1999–2006. Int J Stroke.

[21] Feigin VL (2015). Atlas of the Global Burden of Stroke (1990–2013): the GBD 2013 Study. Neuroepidemiology.

[22] Lu H (2018). Trends in stroke incidence among elderly low-income residents of rural China: a population-based study from 1992 to 2016. Aging.

[23] Institute for Health Metrics and Evaluation (IHME). Epi visualization. 2018. http://vizhub.healthdata.org/epi. Cited 21 May 2021.

[24] Guan W (2019). Time to hospital arrival among patients with acute myocardial infarction in China: a report from China PEACE prospective study. Eur Heart J Qual Care Clin Outcomes.

[25] Xiao H (2018). Impact of the smoke-free legislation on the incidence and mortality of AMI and stroke in Tianjin China: analysis of routinely collected data. Tob Induc Dis.

[26] Bejot Y (2013). Impact of completeness of ascertainment of minor stroke on stroke incidence: implications for ideal study methods. Stroke.

[27] Brown PH, Theoharides C (2009). Health-seeking behavior and hospital choice in China's New Cooperative Medical System. Health Econ.

[28] Lu H (2017). Healthcare seeking behaviour among Chinese elderly. Int J Health Care Qual Assur.

[29] Song Y, Bian Y (2014). Gender differences in the use of health care in China: cross-sectional analysis. Int J Equity Health.

[30] Lewis ME (2016). Estimated incidence and risk factors of sudden unexpected death. Open Heart.

[31] Mehta LS (2016). Acute myocardial infarction in women. Circulation.

[32] Stecker EC (2006). Population-based analysis of sudden cardiac death with and without left ventricular systolic dysfunction: two-year findings from the Oregon Sudden Unexpected Death Study. J Am Coll Cardiol.

[33] Chow C-M (2008). Lack of awareness of heart disease and stroke among Chinese Canadians: results of a pilot study of the Chinese Canadian Cardiovascular Health Project. Can J Cardiol.

[34] Yu AYX (2019). Sex differences in presentation and outcome after an acute transient or minor neurologic event. JAMA Neurol.

[35] Li L, Fu H (2017). China's health care system reform: progress and prospects. Int J Health Plan Manag.

[36] Lambo JA (2011). Completeness of reporting and case ascertainment for neonatal tetanus in rural Pakistan. Int J Infect Dis.

[37] Wang J (2017). Risk factors for stroke in the Chinese population: a systematic review and meta-analysis. J Stroke Cerebrovasc Dis.

[38] Stroke Prevention Project Committee, National Health and Family Planning Commission, and P.R. China (2013). Technical guideline for stroke screening and prevention. Chin J Front Med Sci (Electronic Version).

[39] Wang Y (2018). Is the population detected by screening in china truly at high risk of stroke?. J Stroke Cerebrovasc Dis.

[40] Wang DZ (2017). Analysis on the trends in mortality following acute myocardial infarction from 1999 to 2015 in Tianjin of China. Zhonghua Xin Xue Guan Bing Za Zhi.

[41] Manuel DG (2007). How many people have had a myocardial infarction? Prevalence estimated using historical hospital data. BMC Public Health.

[42] Scarborough P (2016). Assessing the external validity of model-based estimates of the incidence of heart attack in England: a modelling study. BMC Public Health.

