# Supplementary figures and images for: Automatic electronic reporting improved the completeness of AMI and stroke incident surveillance in Tianjin, China: a modeling study
Source: Popul Health Metr. 2023 Feb 6;21:2. doi: 10.1186/s12963-023-00300-2 (PMC9901143; doi:10.1186/s12963-023-00300-2)

AMI

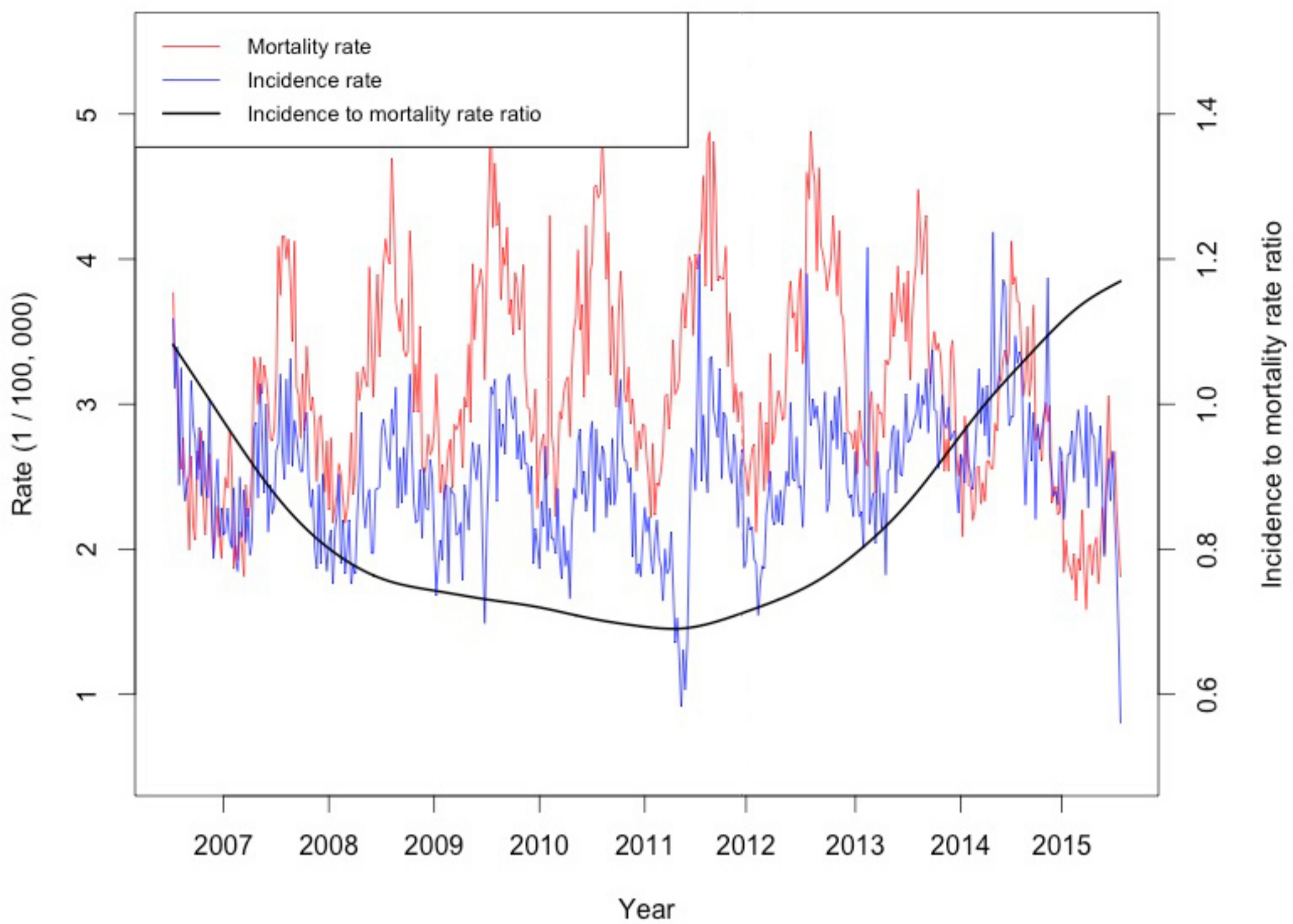

Stroke

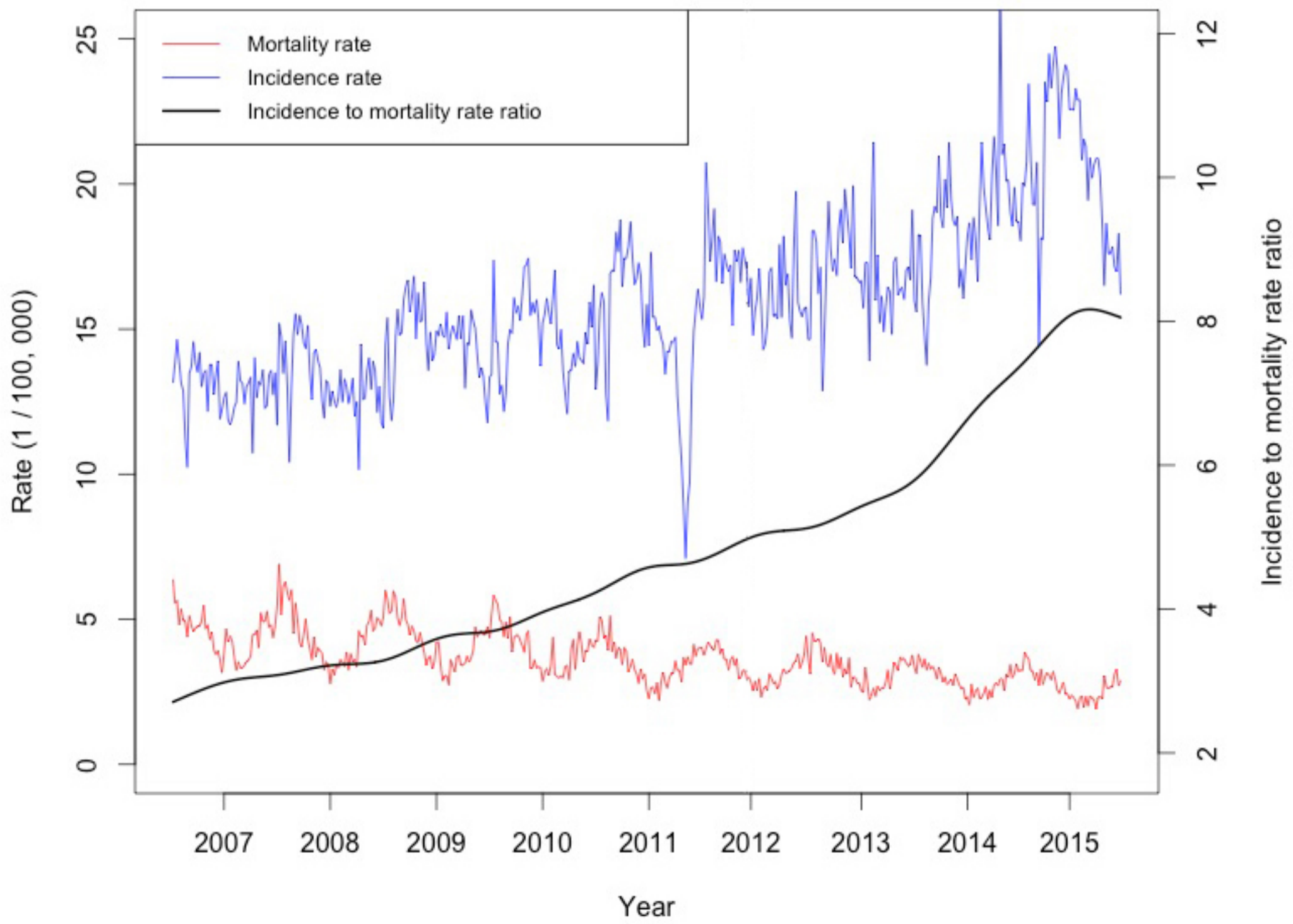

Supplement: Supplementary file 1 — Additional file 1: Figure S1. Trend of AMI and stroke incidence to mortality rate ratio in Tianjin, Age ≥ 35. Red line, mortality rate; Blue line, incidence rate; Black solid line, incidence to mortality rate ratio (smoothed). [file 12963_2023_300_MOESM1_ESM.pdf]
